# Effect of Pets on Human Behavior and Stress in Disaster

**DOI:** 10.3389/fvets.2019.00113

**Published:** 2019-04-18

**Authors:** Aki Tanaka, Jun Saeki, Shin-ichi Hayama, Philip H. Kass

**Affiliations:** ^1^Department of Wildlife Medicine, Nippon Veterinary and Life Science University, Tokyo, Japan; ^2^Department of Population Health and Reproduction, University of California, Davis, Davis, CA, United States; ^3^Department of Veterinary Internal Medicine, Osaka Prefecture University, Osaka, Japan

**Keywords:** pets, animal welfare, disaster, PTSD, evacuation, shelter

## Abstract

Animal-related consequences were not anticipated in disaster preparedness planning in Japan at the time of its massive earthquakes in 2011. Evacuation failure was quite common due to pet ownership in this disaster. Public attention to the welfare of affected animals in this disaster triggered an awareness of the importance of caring for their needs. However, research on human behavior toward pets or effect of pets on human during disasters remains sparse. In this study, post-traumatic stress disorder (PTSD) scores among pet-owners and non-pet owners in Japan's 2011 earthquake disaster were compared, and attitudes toward pets were evaluated. A questionnaire was distributed to attendees, and interviews were performed at an annual animal welfare event. The Japanese-language version of the revised Impact of Event Scale was used to evaluate PTSD from the disaster. PTSD scores were higher in pet-owners compared to non-pet owners immediately after the earthquakes, but were lower in pet-owners compared to non-pet owners 4.4 years following the disaster. Most people opined that pets should evacuate with people, although less than half of non-pet owners agreed with having animals co-located at evacuation centers. In order to enhance safety and security of both humans and animals at evacuation centers, it is important to proactively address animal issues in disaster preparedness planning. Although pets were regarded by some as adverse risk factors for human health and safety during a disaster; this study instead suggests that pets may play an important positive and protective role for disaster victims.

## Introduction

The Great East Japan Earthquakes of March 11th, 2011 were an unprecedented disaster that caused immense damage in a broad area covering approximately 1 million km^2^ (~200 km from east to west in width and ~500 km from north to south in length) along the coastline of northern Japan. The worst affected areas were the Fukushima, Miyagi and Iwate prefectures, where more than 18,300 people were killed or missing. Animals were also severely affected, including ~1,850 cattle, 17,000 pigs, and 2,360,000 chickens that died from the earthquakes and resultant tsunami. Companion animals also fell victim to the disaster, contributing to the deaths of an estimated 16,000 dogs and more than 23,000 cats. The majority of these deaths were in the Miyagi prefecture, accounting for more than 60% of the deaths in dogs and ~90% in cats.

Animal concerns were not included nor clearly defined in disaster preparedness planning in Japan at the time of the earthquakes. With devastating damage impacting people over a broad area, human life remained the top priority for disaster relief immediately following the catastrophe. As a result, pet evacuation and pet-friendly shelters were not widely incorporated as part of the disaster response at the time. More than 2,000 predominantly human-only evacuation shelters were established in Iwate, Miyagi, and Fukushima prefectures; however, pet-friendly shelters accounted for only 3% (65/2151). Acceptance of animals at human evacuation shelters depended on the discretion of shelter's chief operating officer. Some shelters initially allowed animals in the early days of the evacuations, but then ceased accommodating them as the evacuation and sheltering continued due to complaints from some evacuees. Other shelters allowed owners to evacuate with their pets; however, owners were forced to tie or leave them outside in cages, resulting in some being swept away by the floods or frozen to death. Even when pet owners and animals were co-located, some of the former harbored feelings of guilt over creating more emotional distress for other evacuees. Rules to accommodate animals at evacuation shelters were not well-documented nor consistent among shelters, causing conflict between pet owners and non-pet owners. Consequently, many pet owners were unable or reluctant to take refuge at them, remaining instead in their own vehicles in parking lots. Taking shelter in vehicles for prolonged periods imposed different health hazards on pet-owners, including deep-vein thrombosis, with one casualty reported in the Miyagi prefecture.

Consequently, evacuation failure was quite common in pet owners in this disaster. Some people remained in destroyed houses or returned prematurely to save animals, risking their lives. Pets are a recognized risk factor for evacuation failure and prematurely re-entering sites in other natural disasters (1–4); therefore, the need to consider the human-animal bond, and to include animals in evacuation and disaster planning has been promoted (5, 6). In the United States, the Pets Evacuation and Transport Standards Act (PETS) was established after Hurricane Katrina to include companion and service animals in disaster planning. The Act also provided funding to states and localities for the creation, operation, and maintenance of pet-friendly emergency shelters, along with rescuing, caring for, and sheltering animals in an emergency.

Pet ownership in Japan (14.2 and 9.9% of people own dogs and cats, respectively, according to a 2017 report from the Japan Pet Food Association) is not as high as in western countries. However, many pets are still considered family members, as they are in the other countries. This prompted popular and social media to frequently raise questions about the welfare of affected animals in this disaster, and triggered an awareness of elevating the importance animals' needs during disasters, and the urgency of anticipating these concerns for inclusion in disaster preparedness planning for the safety of both animals and people.

Disaster victims suffer a unique and tremendous burden on their mental and physical health (7–10). It is well-known that natural disasters place individuals at substantial risk of post-traumatic stress disorder (11–13), and pet owners are no exception. Although pet ownership has been considered a potential mental health concern during disasters (1), research on human attitudes toward pets or the effect of pets on their owners' emotional states during disasters remain sparse.

The objective of this study was to compare post-traumatic stress disorder scores among pet-owners and non-pet owners at the time of the disaster and ~4 years later. Attitudes toward pets affected by this disaster were also evaluated among disaster victims living in the Miyagi prefecture.

## Materials and Methods

The study population included disaster victims of the Great East Japan Earthquakes of 2011, who continued to reside in the Miyagi Prefecture. It was then sub-divided into four groups: those who owned one or more pets at the time of the earthquakes, those who did not own pets at the time of the earthquakes, those who owned one or more pets 4.4 years after the earthquakes, and those who did not own pets 4.4 years after the earthquakes.

In this descriptive survey study, a questionnaire was distributed to, and interviews were performed with attendees at an annual animal welfare event in Sendai city, Miyagi Prefecture, Japan, on September 22nd, 2015. This annual event was open to the public free of charge and was organized by the city of Sendai, the prefecture of Miyagi, and the local veterinary medical associations. Convenience sampling of attendees of the event was performed by randomly selecting and interviewing participants. Exclusion criteria for the questionnaire were age younger than 12 years old and individuals not experiencing the earthquakes of 2011 in the Miyagi prefecture. Four trained interviewers were recruited and conducted interviews with the attendees until the end of the event. The interviewers received prior training to avoid self-selection and other potential biases. Once the interview was completed for one attendant, the next attendant who entered the entrance of the event site was automatically included in the interview and there was no selection involved. The questionnaire was completely anonymous and did not contain any personal data, including name, address, or medical records. This study was exempt from ethical approval because anonymized datasets were sent to the authors from the Sendai Veterinary Medical Association to perform secondary analyses. A minimum of 200 individuals were sought for participation in order to find a significant difference in the mean PTSD between pet and non-pet owners of at least five scoring units with a common standard deviation of 12.5 scoring units. Using this sample size, with a ratio of 2 pet owners for every non-pet owner sampled, the power to detect a difference in proportions of 0.75 and an odds ratio of 3 was over 90%.

### Demographic Descriptors and Pet-Related Questions

Questions about demographic characteristics of the study population included gender, age, family size, number of pets owned both at the time of the Great East Japan Earthquakes and 4.4 years post-disaster.

### Post-traumatic Stress Disorder (PTSD) Scores

The Japanese-language version of the Impact of Event Scale—Revised (IES-R-J) (14) was used to evaluate PTSD from the Great East Japan Earthquakes. This questionnaire consisted of 22 questions in three subscales, including intrusion items, avoidance items, and hyperarousal items, and utilized five-point scales (0–4) for each question, leading to development of a composite PTSD score. A cutoff value of 24/25 in total score was used to define clinically concerning PTSD and partial PTSD (14). The respondents were asked to answer how strong (0–4 scale) they recalled feeling about items in the questionnaire 1 month after the disaster and at the time of the event (i.e., 4.4 years after the disaster).

### Evacuation and Pets

The following questions were asked to evaluate attitudes toward pets during disasters: (1) Do you think pets should evacuate with owners at the time of disaster (yes/no); (2) Do you think pets should stay in the same room with owners at shelters (yes/no); (3) Would you feel nervous if animals were in the same room at a shelter (yes/no); (4) Why would you feel nervous (open answer)?

### Statistical Analysis

The Shapiro-Wilk test was performed to evaluate normality of data. Analysis of variance was performed to analyze the relationship between pet ownership at the time of the disaster and 4.4 years later on PTSD; the dependent variable was PTSD score, and the independent variable was pet ownership (disaster victims who owned pets versus those who did not own pet). Logistic regression was performed to analyze the association between pet ownership status and whether pets should be evacuated with people, whether pets could stay in proximity with humans, and whether animals were nuisances. The Mann-Whitney test was performed to investigate the difference of median scores for each PTSD symptoms (intrusion, avoidance, and hyperarousal) in dog owners at the time of disaster and 4.4 years after.

Stata/IC 13.1 (StataCorp, College Station, Texas, USA) was used for all analyses. For statistical inferences, two-sided hypothesis tests were used with a 5% significance level.

## Results

There were 216 respondents who answered the questionnaire and were interviewed at the event: their characteristics are described in Table 1.

**Table 1:** Characteristics of the respondents (*n* = 216) conducted at an animal welfare event in the city of Sendai, September 22nd, 2015.

	**Number of respondents (%)**
**VARIABLES**	
**SEX**	
Female	138 (67.0)
Male	68 (33.0)
**AGE**	
13–19 years	8 (3.7)
20–29 years	21 (9.7)
30–39 years	39 (18.1)
40–49 years	62 (28.7)
50–59 years	50 (23.2)
60–69 years	24 (11.1)
70–79 years	9 (4.2)
80–89 years	3 (1.4)
**FAMILY STRUCTURE**	
Single family	20 (9.8)
Families with adults only	127 (62.0)
Families with younger children	51 (24.9)
Families with members needing nursing care	7 (3.4)
**PET OWNERSHIP AT THE TIME OF DISASTER**	
Owned one or more pets	101 (46.8)
Did not own pets	115 (53.2)
Of those who owned pets, dogs	69 (68.3)
Cats	22 (21.8)
Dogs and cats	9 (8.9)
Other animals such as gold fish, hamsters, turtles, and birds	10 (9.9)
Unknown pet	1 (1.0)
**PET OWNERSHIP AT 2015**	
Owned one or more pets	136 (63.0)
Did not own pets	80 (37.0)
Of those who owned pets, dogs	78 (57.4)
Cats	21 (15.4)
Dogs and cats	14 (10.3)
Other animals such as gold fish, hamsters, turtles, and birds	16 (11.8)

### PTSD Scores

The median overall PTSD score 1 month after the disaster was 26 (range 1–83) (*n* = 202) and was significantly higher than the median PTSD score of 15 reflecting emotions 4.4 years after the disaster (range 1–78) (*n* = 155) (*p* < 0.0001). The median PTSD scores of pet owners and non-pet owners at 1 month after and 4.4 years after the disaster are described in Table 2.

**Table 2:** Median PTSD scores (range) for pet owners and non-pet owners in the city of Sendai, Japan 1 month after the Great East Japan Earthquakes of 2011 (*n* = 204) and 4.4 years after the Great East Japan Earthquakes of 2011 (*n* = 216).

		***n***	**Median PTSD score (range)**	***p*-value**
1 month after	Pet owners	110	27 (1–81)	0.337
	Non-pet owners	94	24 (1–83)	
4.4-year after	Pet owners	136	13 (1–52)	0.0381
	Non-pet owners	80	20 (3–78)	

Pet ownership did not have an association with PTSD score 1 month after the disaster (*p* = 0.337). However, when pet type was disaggregated to specific animal species, dog owners had significantly higher PTSD scores than non-pet owners (*p* = 0.025) (Table 3). The median PTSD score (range) for dog owners 1 month following the disaster was 30 (4–81), for cat owners was 29 (1–57), for both dog and cat owners was 19 (16–32), and for other animal owners was 16 (3–55).

**Table 3:** Relationship of pet ownership (dog, cat, dog and cat, and other animals) on PTSD score 1 month after the Great East Japan Earthquakes for pet owners and non-pet owners in the city of Sendai, Japan (*n* = 204).

		***n***	**regression coefficient**	**95% CI**	***p*-value**
PTSD score 1 month after	Non-pet owner	94	Reference		
	Dog owner	69	6.9	0.9–12.9	0.025
	Cat owner	22	−1.2	−10.3–7.7	0.791
	Dog and cat owner	9	−7.6	−21.4–6.1	0.276
	Other animals	10	−7.4	−20.4–5.7	0.266

*95% CI, 95% confidence interval of the regression coefficient. P-values refer to Ho: regression coefficient, 0*.

Pet owners had lower PTSD scores 4.4 years after the disaster than non-pet owners (*p* = 0.0035) (Table 4). When the pets were disaggregated to specific animals, PTSD scores for owners of other animals, such as fish, hamsters, turtles and birds, were significantly lower than for non-pet owners (*p* = 0.041) (Table 5). The median PTSD score (range) for dog owners 4.4 years after the disaster was 14 (1–78), for cat owners was 20 (4–41), for both dog and cat owners was 5 (3–24), and for other animals' owners was 14 (3–27). Each median score of PTSD symptom (intrusion, avoidance, and hyperarousal) in dog owners were significantly greater at the time of disaster than 4.4 years after (Table 6).

**Table 4:** Relationship of pet ownership on PTSD score 4.4-year after the Great East Japan Earthquakes of 2011 for pet owners (*n* = 80) and non-pet owners (*n* = 136) in city of Sendai, Japan.

		***n***	**Regression coefficient**	**95% CI**	***p*-value**
PTSD score 4.4 years after	Non-pet owner	80	Reference		
	Pet owner	136	−5.5	−10.6 to −0.4	0.035

*95% CI, 95% confidence interval of the regression coefficient. P-values refer to Ho: regression coefficient, 0*.

**Table 5:** Association of pet ownership (dog, cat, dog and cat, and other animal) on PTSD score 4.4 years after the Great East Japan Earthquakes for pet owners and non-pet owners in city of Sendai, Japan (*n* = 202).

		***n***	**regression coefficient**	**95% CI**	***p*-value**
PTSD score 4.4 years after	Non-pet owner	80	reference		
	Dog owner	78	−3.5	−9.2–2.3	0.235
	Cat owner	21	−5.6	−14.2–3.1	0.206
	Dog and cat owner	14	−5.6	−17.2–3.6	0.2
	Other animal	9	−5.6	−23.4–0.5	0.041

*95% CI, 95% confidence interval of the regression coefficient. P-values refer to Ho: regression coefficient, 0*.

**Table 6:** Median scores (range) for each aspect of PTSD symptom (intrusion, avoidance and hyperarousal) at the time of disaster (*n* = 85) and 4.4 years after (*n* = 79) in dog owners.

	**Median score (range)**		
**PTSD symptom**	**At the time of disaster (*n* = 85)**	**4.4 years after (*n* = 79)**	***p*-value**
Intrusion	11.5 (0–32)	4 (0–19)	*p* < 0.0001
Avoidance	10 (0–29)	4 (0–28)	*p* < 0.0001
Hyperarousal	6 (0–23)	3 (0–21)	*p* < 0.0001

### Evacuation and Pets

**Figure 1:**
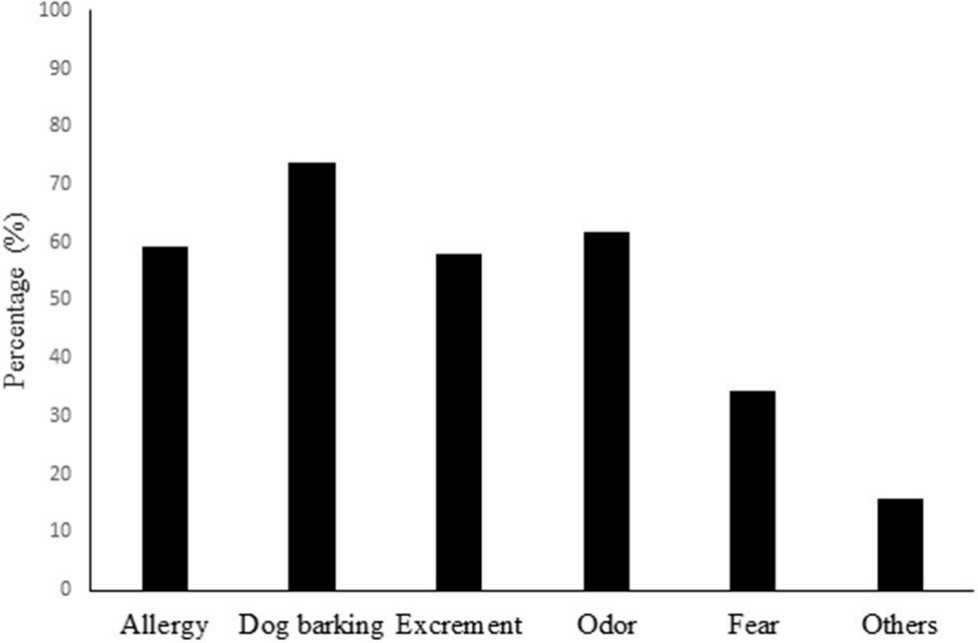
Reasons for sense of aversion to animals at evacuation shelters in disaster victims in city of Sendai, Japan, 2015 (*n* = 77).

95.6% (130/1365) of pet owners, in contrast to 72.5% (58/80) of non-pet owners expressed the opinion that pets should be evacuated with people in the face of disaster. 73.1% (98/134) of pet owners, in contrast to 45.6% (36/79) of non-pet owners considered that pets should be co-located with people in the evacuation center. 37.0% (50/135) of pet owners and 32.5% (26/80) of non-pet owners answered that they would feel nervous if animals stayed in proximity to them at the evacuation shelter. The reasons for their nervousness toward animals included allergy, dog barking, excrement, odor, fear, infectious disease, and others (Figure 1). Pet ownership was significantly associated with attitude toward pet evacuation (*p* < 0.0001) or co-location of animals in the evacuation center (*p* < 0.0001); however, it was not significantly associated with a sense of aversion when animals were in proximity at the evacuation center (*p* = 0.477) (Table 7). There were no significant differences between the different types of pet-owners for attitudes toward pet evacuation, co-location, nor a sense of aversion (*p* = 0.079, 0.10, and 0.080, respectively).

**Table 7:** Logistic regression analysis of whether a pet should (vs. should not) evacuate with people (*n* = 203), pet should (vs. should not) co-locate with people (*n* = 198), and having a sense of aversion (vs. not) to pets in shelters (*n* = 212) for pet owners and non-pet owners in the city of Sendai, Japan, 2015.

	***n***	**Odds ratio**	**95% CI**	***p*-value**
**PETS SHOULD EVACUATE WITH PEOPLE**				
Non-pet owners	68	Reference		
Pet owners	135	3.4	1.0–11.1	0.042
**PETS SHOULD CO-LOCATE WITH PEOPLE**				
Non-pet owners	65	Reference		
Pet owners	133	3.3	1.8–5.9	<0.0001
**SENSE OF AVERSION TO PETS**				
Non-pet owners	79	Reference		
Pet owners	133	1.2	0.7–2.3	0.477

*95% CI, 95% confidence interval of the odds ratio. P-values refer to Ho: odds ratio, 0*.

## Discussion

The importance of incorporating animal components into disaster planning has been supported by considerable evidence (1, 3, 5, 15–18). Considerations for accommodating animals included economics, public health, emotional well-being of humans, and animal welfare (19–23). These four areas overlap, and the latter two have been increasingly prominent by more recent acceptance of the scientific basis of the human animal bond. People feel disturbed to see distressed animals suffering in disasters and people would risk their lives to save animals even they were not owned by them (15, 24). Animals have been considered adverse risk factors in disasters for human survival due to animal attachment and post-disaster distress due to animal loss (25, 26). However, recent studies suggest that animal attachment can be a protective factor that promotes survival and facilitates recovery (16, 27–29). Animals could play an important role in increasing disaster resilience by if public health and emergency officials disseminate disaster preparedness and planning information to vulnerable people, including those experiencing social isolation and those nervous around strangers, facilitate better communication; provide better motivation for vulnerable people to prepare and act; and facilitate recovery by avoiding animal loss and animal-assisted interactions (27).

In this study, PTSD score was investigated in the city of Sendai to evaluate the effect of pets on emotional state in disaster recovery. PTSD score uses a self-rating scale to measure stress responses after traumatic events. In this study, the Japanese-language version of the Impact of Event Scale-Revised (IES-R-J) was employed, which has been used to assess workers with lifetime mixed traumatic events, survivors of an arsenic poisoning case, survivors of the Hanshin-Awaji earthquake, and survivors of the Tokyo Metro sarin attack to investigate its reliability and validity (14). It has been shown to have high retest reliability and good internal consistency through its high values of Cronbach's coefficient alpha across the whole scale and subscales (14).

PTSD score was significantly higher 1 month after the earthquakes compared to 4.4 years later. During the acute phase, in the chaotic aftermath of the earthquakes when infrastructure was disrupted, a number of the disaster victims were evacuated to shelters in the Miyagi prefecture. Even those not displaced from their home also experienced tremendous psychological distress caused by this unprecedented disaster. Dog owners had significantly higher PTSD scores compared to non-pet owners during this acute phase. For disaster victims, already distressed under a very traumatic situation, dog ownership might have been perceived as an additional burden. Having dogs as pets requires more care compared to cats, birds, hamsters, or goldfish due to the additional need of physical activity and exercise (e.g., dog-walking), and this extra commitment may had been the reason why dog owners had higher PTSD scores compared to other pet owners. Many pet owners were prohibited from accommodating animals at emergency evacuation centers, and struggled to take initiatives by themselves (30), which might have added to an already substantial emotional burden. Such owners may have felt even more stressed they became aware of their lack of preparedness about the canine members of their family when they were already experiencing other losses in the midst of disaster. PTSD scores among rescue workers, fire fighters, DMAT members, local disaster relief and reconstruction works have been reported (31–34); however, there were no PTSD scores among general disaster victims using the same instrument in this study.

After 4.4 years, PTSD scores were significantly higher for non-pet owners compared to pet owners. In the ensuing 4.4 years following the disaster, reconstruction has begun, and the affected area (including the city of Sendai) is gradually returning to normal. Pet ownership may have had a positive effect on recovery for disaster victims to help overcome their distress, as the benefits of pets on physical and mental health of humans are well-documented. For example, dog and cat owners in general made fewer doctor visits and took less medication for heart problems and sleeping difficulties than non-pet owners (35). Pet ownership also had a significant positive effect on modifying social support and change in psychological well-being (35, 36). Interaction with animals has been associated with mitigating PTSD from sexual assault (37, 38) and had positive effects on reduction in anxiety (39), loneliness (40) and agitated behavior (41). Disaster victims suffer from tremendous PTSD and depression (11), as many of them are displaced from their homes and separated from familiar surroundings. Evacuation at shelters and temporary housing causes social isolation for people; however, pro-social behaviors with animals (42) and dog walking could be beneficial to prevent loneliness and enhance social interaction (43) for disaster victims.

Animals in this disaster attracted massive attention from the public and media. Tragic situations of animals killed or injured from the tsunami and animals left behind in the restricted area in Fukushima prefecture were repeatedly shown on TV and reported. Even with relatively low pet-ownership in Japan, animal issues in disasters gained great public attention, and this study revealed that non-pet owners also cared about animals. The equivalent of over three million U.S. dollars were donated for rescuing and sheltering dogs and cats. Following Japan's experience with this disaster, public sentiment shifted, and ignoring affected animals during disasters was no longer politically tolerable. Policy implications of these public concerns has now resulted in consideration of pet evacuation being introduced into government disaster preparedness planning.

This study revealed that almost all surveyed pet owners believed that animals should be evacuated, implying that pet owners will evacuate with their animals during disasters whether a shelter is pet-friendly or not, and which could affect their decision to not evacuate if they feel they cannot safely do so with their pet. It has been shown elsewhere that attachment to animals and closeness to pets could interfere with action plans in disaster (15, 16). The majority of non-pet owners also believed that animals should not be left behind. However, if the animals were to share space with people at a shelter, more than half of the non-pet owners would have been opposed. 32–37% of people potentially felt nervous when animals were around them, whether they had pets or not. Because pet ownership is low in Japan, but pet evacuation was encouraged, it is important to plan for this eventuality to assure safety and security for both people and animals. Life for evacuees is stressful, and if animals became a nuisance, it would add extra stress to both pet owners and non-pet owners at an evacuation shelter. It had even been found that some pet owners develop a sense of anxiety in the presence of other animals. All evacuation shelters, whether they allow pets or not, should have a plan for housing animals appropriately to avoid confusion and improve welfare in the midst of an emergency for both animals and people.

Limitations of this study included convenience sampling of respondents and interviewees. Four interviewers were recruited and performed interviews throughout the event; however, not all the attendees could be included in the study. Data were collected from attendants of an animal welfare event in the city of Sendai, so the attendants might have already been cognizant of animal welfare and issues of pets during disasters, whether they owned pets or not. This tendency might have also affected their attachment and commitment toward pets, which could have influenced their PTSD response during the disaster. Another limitation might be recall bias: the respondents were asked to recall their individual feelings at the time of the 2011 earthquakes. However, many respondents stated that they would never forget their feelings that precedented the earthquakes, so their memories might be relatively sound and valid. Finally, pets owned were mostly dogs and cats, so it was difficult to evaluate the effects of owners of other species due to the small sample sizes.

Pets were regarded as adverse risk factors for human health and safety during the disaster; however, this study suggest pets may instead play an important role as a protective factor for disaster victims. Pets may be beneficial for disaster victims during the recovery phase to cope with PTSD. In order to enhance safety and security of both humans and animals at evacuation centers, it is important to address animal issues and include animal considerations in disaster preparedness planning in advance. Governmental animal management practices have started to gradually change, such as promoting evacuation shelters to be more pet-friendly, standardizing animal sheltering procedures in disasters, and providing more training opportunities for disaster planning at the government level. Veterinary Medical Assistance Teams (VMAT) have been launched through the Japan Veterinary Medical Association and started to provide training opportunities to veterinary professionals. Multidisciplinary coordination that includes veterinarians, fire departments, police departments, and human health care professionals is integral for both animal and human safety in disasters.

## Ethics Statement

This study included use of a questionnaire from an annual animal event in the city of Sendai, Japan. The questionnaire was completely anonymously and did not contain any personal data, including name, address, health status, or medical records. This study was exempt from ethical approval because anonymized datasets were sent to the authors from a third party (the Sendai City Veterinary Medical Association) to perform secondary analyses.

## Author Contributions

AT contributed to the conception and design of the study, the acquisition, analysis and interpretation of data, software, and writing original draft, review and editing. PK contributed to validation, writing, review, and editing. JS contributed to the conception and design of the study, review and editing. SH contributed to the conception and design of the study, supervision, project administration, funding acquisition, review, and editing.

### Conflict of Interest Statement

The authors declare that the research was conducted in the absence of any commercial or financial relationships that could be construed as a potential conflict of interest.
